# Impact of *COL6A4P2* gene polymorphisms on the risk of lung cancer: A case-control study

**DOI:** 10.1371/journal.pone.0252082

**Published:** 2021-05-21

**Authors:** Xiaodong Dang, Wenhui Zhao, Chen Li, Hua Yang, Dianzhen Li, Shanshan Zhang, Tianbo Jin

**Affiliations:** 1 Department of Anesthesiology, Shaanxi Provincial Cancer Hospital, Shaanxi, Xi’an, China; 2 Xi’an 21st Century Biological Sicence and Technology Co., Ltd, Shaanxi, Xi’an, China; 3 Key Laboratory of Molecular Mechanism and Intervention Research for Plateau Diseases of Tibet Autonomous Region, School of Medicine, Xizang Minzu University, Xianyang, Shaanxi, China; 4 Key Laboratory of Resource Biology and Biotechnology in Western China (Northwest University), Ministry of Education, School of Life Sciences, Northwest University, Shaanxi, Xi’an, China; Suez Canal University Faculty of Medicine, EGYPT

## Abstract

Lung cancer (LC) is a malignant tumor that poses the greatest threat to human health and life. Most studies suggested that the occurrence of LC is associated with environmental and genetic factors. We aimed to explore the association between *COL6A4P2* single nucleotide polymorphisms (SNPs) and CHD risk in the Chinese Southern Han population. Based on the ‘case-control’ experimental design (510 cases and 495 controls), we conducted an association study between five candidate *COL6A4P2* SNPs and the corresponding LC risk. Odds ratio (OR) and 95% confidence intervals (CIs) were calculated by logistic regression to analyze the LC susceptibility under different genetic models. The results showed that *COL6A4P2* rs34445363 was significantly associated with LC risk under alleles model (OR = 1.26, 95%CI: 1.01–1.58, *p* = 0.038). In addition, rs34445363 was also significantly associated with LC risk under the log-additive model (OR = 1.26, 95%CI: 1.01–1.58, *p* = 0.041). The results of subgroup analysis showed that rs34445363 (OR = 1.42, 95%CI: 1.03–1.95, p = 0.033) and rs61733464 (OR = 0.72, 95%CI: 0.52–0.99, *p* = 0.048) were both significantly associated with LC risk in the log-additive model among participants who were ≤ 61 years old. We also found that the variation of rs34445363 (GA vs. GG, OR = 1.73, 95%CI: 1.04–2.86, *p* = 0.034) and rs77941834 (TA vs. TT, OR = 1.88, 95%CI: 1.06–3.34, *p* = 0.032) were associated with LC risk in the codominant model among female participants. Our study is the first to find that *COL6A4P2* gene polymorphism is associated with LC risk in the Chinese Han population. Our study provides a basic reference for individualized LC prevention.

## Introduction

Lung cancer (LC) is a malignant tumor with the fastest growth in morbidity and mortality and the greatest threat to human health and life [1]. According to the Global Cancer Observatory database (http://gco.iarc.fr/) [2], there are 2,093,876 new cases of LC worldwide, accounting for 11.6% of all cancers; the number of people who died of LC in 2018 is 1,761,007, accounting for 17.9% of all cancer deaths in 2018. Among them, the incidence and mortality of LC in women were 13.1% and 6.9%, respectively. LC has become the most malignant tumor with the highest incidence and mortality [3–5]. In China, LC also has a high incidence and mortality, and its morbidity and mortality in men are more than twice that of women [6]. Most studies have suggested that the occurrence of LC is associated with environmental (smoke, occupational exposure, and air pollution) and genetic factors [7, 8]. In particular, genetic factors play an essential role in the occurrence of LC. Li *et al*. [9] revealed that LC susceptibility in the Chinese Han population was associated with *HOTAIR* gene mutations. Dimitrakopoulos *et al*. [10] believed that the *NF-kB2* gene mutation is significantly associated with LC risk. However, the association between *COL6A4P2* gene polymorphisms and LC susceptibility has not been reported.

*COL6A4P2* (collagen type VI alpha 4 pseudogene 2), also named *COL6A4*, is located on Chr.3q22 in humans. *COL6A4* expresses type VI collagen (COL6), an extracellular matrix protein that plays a vital role in maintaining lung tissue integrity. Chiu *et al*. [11] showed by quantitative secretion cleavage that *COL6* is a protein involved in tumor metastasis. Voiles *et al*. [12] demonstrated that the expression of the *COL6* protein in LC is upregulated. Thus, we suspect that *COL6A4* may be associated with LC.

It has been reported that *COL6A4* is an unprocessed pseudogene due to the presence of multiple stop codons in the gene sequence [13]. Many studies have shown that pseudogenes play an essential role in cancer development. Cheng *et al*. [14] have found that pseudogenes affect the occurrence and development of cancer by forming lncRNA-pseudogene-mRNA competitive triples. Lynn *et al*. [15] have confirmed that polymorphisms in the *MYLKP1* pseudogene is associated with an increased risk of colon cancer. Wei *et al*. [16] have found that the pseudogene *DUXAP10* promotes the invasiveness of LCs. Therefore, we speculated that the *COL6A4P2* gene minght play a role in cancer development.

In this study, we first explored the association between the *COL6A4P2* gene and LC risk, revealing the association between *COL6A4P2* gene polymorphism and LC susceptibility in the Chinese Han population.

## Materials and methods

### Study participants

Using a case-control design, 510 LC patients (mean age: 60.78 ± 9.96 years) and 495 controls (mean age: 61.94 ± 7.72 years) were enrolled in the study. All patients were recruited from Shaanxi Provincial Cancer Hospital. Patient inclusion criteria were as follow: 1) newly diagnosed LC, 2) histopathological LC diagnosed by an experienced pathologist, 3) no previous radiation therapy or chemical therapy, and 4) no history of cancer or metastatic carcinoma. Patients with asthma, bronchitis, pneumonia, lung abscess, tuberculosis, other lung diseases, autoimmune diseases, trauma or other tumors were excluded from the study. After that, we investigated and collected information regarding clinical indicators of LC patients, including sex, age, histological classification, tumor stage, and the status of lymph node metastasis.

The controls were healthy volunteers recruited from the Shaanxi Provincial Cancer Hospital during the same period. No medical or family history of cancer or any pulmonary disease was the inclusion criteria for the control group. At the time of recruitment, trained personal interviewed using a structured questionnaire to obtain information regarding their demographic characteristics.

### Data collection

This study was approved by the Shaanxi Provincial Cancer Hospital ethics committee and conformed to the ethical principles for medical research involving humans of the World Medical Association Declaration of Helsinki. All participants signed informed consent forms before participating in the study. Subsequently, a sample of approximately 5 mL of venous blood was obtained from each participant and collected into tubes containing ethylenediaminetetraacetic acid for anticoagulation. Genomic DNA was extracted from peripheral blood samples using a whole-blood genomic DNA extraction kit (GOLDMAG, Xi´an, China) according to the manufacturer’s instructions. The purity and concentration of the DNA samples were evaluated using a NanoDrop 2000C system (Thermo Scientific, Waltham, MA, USA). Isolated DNA was stored at −80°C until analysis.

### SNP genotyping

Five candidate SNPs in the *COL6A4P2* gene were selected with a minor allele frequency (MAF) > 0.05 from the global population in the 1,000 Genome Projects (http://www.internationalgenome.org/). We then used HaploReg v4.1 (https://pubs.broadinstitute.org/mammals/haploreg/haploreg.php) to predict the possible functions of the SNPs. The primers for amplification and single-base extension were designed using the Assay Design Suite, V2.0 (https://agenacx.com/online-tools/). Genotyping of the five SNPs was carried out on the MassARRAY iPLEX (Agena Bioscience, San Diego, CA, USA) platform using matrix-assisted laser desorption ionization–time of flight mass spectrometry [17]. Genotyping results were generated using Agena Bioscience TYPER software, version 4.0. Genotyping was performed by laboratory personnel in a double-blinded manner.

### Analysis of COL6A4P2 and SNPs expression

Data regarding the expression of *COL6A4P2* in LC were obtained from the UALCAN online database (http://ualcan.path.uab.edu/analysis.html), a web server that provides customizable functions. Tumors and normal samples in the UALCAN database were derived from The Cancer Genome Atlas (TCGA) and the Genotype-Tissue Expression (GTEx) projects. The effect of *COL6A4P2* gene expression on LC prognosis was predicted using the OncoLnc database (http://www.oncolnc.org/). We also predicted the expression of SNPs in the *COL6A4P2* gene in normal lung tissues using the GTEx database (https://gtexportal.org/home/).

### Statistical analyses

An independent sample t-test was used to assess differences in the population and clinical characteristics of the study participants. Fisher’s exact tests for HWE were performed by comparing the observed and expected genotype frequencies to calculate the genotype frequencies among the controls. Pearson’s χ^2^ test was used to compare the allelic and genotype frequencies of each SNP between LC patients and controls. Multiple genetic model analyses (codominant, dominant, recessive, and log-additive) were performed using PLINK software (http://zzz.bwh.harvard.edu/plink/ld.shtml) to assess the association between SNPs and LC risk. Furthermore, we calculated stratification factors using sex and age to adjust for possible confounders. Finally, we used Haploview software (version4.2) to construct haplotypes and to estimate the pairwise linkage disequilibrium using the SHEsis software platform (http://analysis.bio-x.cn/myAnalysis.php) was used to estimate the association between haplotype and LC risk. Odds ratios (ORs) and 95% confidence intervals (CIs) were calculated using logistic regression analyses adjusted for sex and age [18], with the wild-type allele used as a reference. Statistical analyses were performed using SPSS software (version 21.0, IBM Corporation, Armonk, NY, USA). All *p*-values of statistical tests were two-sided, and *p* < 0.05, which considered indicative of statistical significance. We also conducted a false-positive report probability (FPRP) analysis to detect whether the significant findings were just chance or noteworthy observations [19].

## Results

### Characteristics of cases and controls

The basic clinical information of patients with LC and controls is shown in Table 1. Five hundred and ten patients were presented with a different distribution, according to age (age ≤ 61, 266 cases; age > 61, 244 cases), gender (male, 355 cases; female, 155 cases), pathological type (lung squamous cell carcinoma [LUSC], 120 patients; lung adenocarcinoma [LUAD], 188 patients), tumor stage (Ⅰ-Ⅱ, 84 cases; Ⅲ-Ⅳ, 261 cases), and lymph node metastasis (LNM) status (positive, 215 cases; negative, 84 cases).

**Table 1:** The comparison of basic characteristics between cases and controls.

Characteristics		Case (n = 510)	Control (n = 495)
Age	≤ 61	266	224
	> 61	244	271
	Mean ± SD	60.78 ± 9.96	61.94 ± 7.72
Gender	Male	355	346
	Female	155	149
Pathological type	LUSC	120	
	LUAD	188	
	Unknown	202	
Tumor stage	Ⅰ-Ⅱ	84	
	Ⅲ-Ⅳ	261	
	Unknown		
LNM	Positive	215	
	Negative	84	
	Unknown		

LUSC = lung squamous cell carcinoma; LUAD = lung adenocarcinoma; LNM = lymph node metastasis.

Basic information and allele frequencies of *COL6A4P2* gene polymorphisms are presented in Table 2. The genotype distribution of all SNPs in the control subjects met the HWE (*p* > 0.05). HaploReg function annotation results revealed that SNPs associated with LC risk were successfully predicted to have biological functions. The association between *COL6A4P2* polymorphisms and LC risk under the allele model is shown in Table 2, and the results showed that rs34445363 was associated with an increased LC risk (OR = 1.26, 95%CI: 1.01–1.58, *p* = 0.038), and there were no differences between the other four SNPs (rs7625942, rs77941834, rs61733464, and rs11914893) in the *COL6A4P2* gene and LC risk (*p* > 0.05).

**Table 2:** Basic information about SNPs in COL6A4P2 and association with risk of lung cancer in allele model.

Gene	SNP ID	Chr.	Alleles(A/B)	Frequency (MAF)		*p*-value for HWE	OR (95% CI)	*p*	Function
				Case	Control				
COL6A4P2	rs34445363	3q22.1	A/G	0.217	0.180	0.879	**1.26 (1.01–1.58)**	**0.038**	Selected eQTL hits
COL6A4P2	rs7625942	3q22.1	A/G	0.223	0.225	0.608	0.98 (0.80–1.21)	0.915	Motifs changed, Selected eQTL hits
COL6A4P2	rs77941834	3q22.1	A/T	0.122	0.097	0.798	1.29 (0.97–1.71)	0.086	Motifs changed, Selected eQTL hits
COL6A4P2	rs61733464	3q22.1	A/G	0.186	0.213	0.346	0.85 (0.68–1.06)	0.146	DNAse, Motifs changed, Selected eQTL hits
COL6A4P2	rs11914893	3q22.1	A/C	0.108	0.115	0.825	0.93 (0.70–1.23)	0.620	Motifs changed, GRASP QTL hits

SNP = single nucleotide polymorphism; Chr. = chromosome; A/B = minor/major, MAF = minor allele frequency; HWE = Hardy Weinberg equilibrium.

*p* < 0.05 indicates statistical significance.

Bold values indicate a significant difference.

### Association between the COL6A4P2 gene and the risk of LC

Genetic models (codominant, dominant, recessive, and log-additive) and genotype frequencies were used to identify any associations between the SNPs and the risk of LC. The results showed that rs34445363 in the *COL6A4P2* gene significantly increased the risk of LC in the log-additive model (adjusted for age and sex, OR = 1.26, 95%CI: 1.01–1.58, *p* = 0.041, Table 3), and no significant difference was found for the other SNPs between cases and controls (all *p* > 0.05).

**Table 3:** Distribution of genotypes of COL6A4P2 polymorphism depicting their association with lung cancer risk and its histological subtypes.

SNP ID	Model	Genotype	Control	LC			LSCC			LUAD		
				Case	OR (95%CI)	*p*	Case	OR (95%CI)	*p*	Case	OR (95%CI)	*p*
rs34445363	Codominant	GG	329	313	1.00		72	1.00		112	1.00	
		GA	146	173	1.25(0.10–1.64)	0.102	43	1.27(0.82–1.96)	0.278	66	1.39(0.96–2.00)	0.082
		AA	15	24	1.63(0.84–3.17)	0.151	5	1.52(0.52–4.46)	0.442	10	1.88(0.81–4.36)	0.144
	Dominant	GG	329	313	1.00		72	1.00		112	1.00	
		GA/AA	161	197	1.29(0.99–1.67)	0.056	48	1.29(0.85–1.97)	0.229	76	**1.43(1.01–2.04)**	**0.046**
	Recessive	GG/GA	475	486	1.00		115	1.00		178	1.00	
		AA	15	24	1.51(0.78–2.92)	0.220	5	1.40(0.48–4.06)	0.533	10	1.68(0.73–3.87)	0.223
	Log-additive	--	--	--	**1.26(1.01–1.58)**	**0.041**	--	1.26(0.88–1.80)	0.212	--	**1.38(1.02–1.86)**	**0.034**
rs61733464	Codominant	GG	310	340	1.00		82	1.00		133	1.00	
		GA	158	150	0.86(0.66–1.13)	0.278	33	0.79(0.50–1.24)	0.299	46	**0.65(0.44–0.96)**	**0.031**
		AA	26	20	0.70(0.38–1.28)	0.246	5	0.75(0.27–2.07)	0.581	9	0.76(0.34–1.69)	0.504
	Dominant	GG	310	340	1.00		82	1.00		133	1.00	
		GA/AA	184	170	0.84(0.65–1.09)	0.181	38	0.78(0.51–1.21)	0.265	55	**0.66(0.46–0.96)**	**0.031**
	Recessive	GG/GA	468	490	1.00		115	1.00		179	1.00	
		AA	26	20	0.73(0.40–1.34)	0.310	5	0.81(0.30–2.21)	0.683	9	0.87(0.39–1.91)	0.724
	Log-additive	--	--	--	0.85(0.68–1.05)	0.139	--	0.82(0.57–1.18)	0.285	--	0.74(0.55–1.01)	0.059

SNP = single nucleotide polymorphism; LC = lung cancer; LUAD = lung adenocarcinoma; LSCC = lung squamous cell carcinoma; OR = odds ratio; 95%CI = 95% confidence interval.

*p* < 0.05 indicates statistical significance.

Bold values indicate a significant difference.

Furthermore, we identified by pathological analysis that rs34445363 locus variation significantly increased the risk of LUAD in the dominant model (adjusted by age and gender, GA/AA vs. GG, OR = 1.43, 95%CI: 1.01–2.04, *p* = 0.046) and log-additive model (adjusted by age and gender, OR = 1.38, 95%CI: 1.02–1.86, *p* = 0.034); However, mutations of rs61733464 in the *COL6A4P2* gene have a lower incidence of LUAD with the GA genotype in the codominant model (adjusted by age and gender, GA vs. GG, OR = 0.65, 95%CI: 0.44–0.96, *p* = 0.031) and under the dominant model (adjusted by age and gender, GA/AA vs. GG, OR = 0.66, 95%CI: 0.46–0.96, *p* = 0.031).

### Association between COL6A4P2 polymorphism and clinicopathological features

To evaluate the association of *COL6A4P2* SNPs with various clinicopathological features, we segregated patients according to the clinical stage (I–II vs. III–IV) and LNM status (positive vs. negative). There was no significant association between LNM status and *COL6A4P2* polymorphism variation (S1 Table). However, for the rs77941834 variant, the codominant model (adjusted by age and gender, TA vs. TT, OR = 0.52, 95%CI: 0.29–0.94, *p* = 0.030), dominant model (adjusted by age and gender, TA/AA vs. TT, OR = 0.49, 95%CI: 0.28–0.86, *p* = 0.013), and log-additive model (adjusted by age and gender, OR = 0.55, 95%CI: 0.34–0.87, *p* = 0.011) significantly decreased the LC risk in patients with III-IV as compared to patients with I-II tumor stage (Table 4). No statistically significant association was observed for tumor staging and the other four SNPs (rs34445363, rs7625942, rs61733464 and rs11914893).

**Table 4:** Association between COL6A4P2 polymorphism and tumor staging of lung cancer.

SNP ID	Model	Genotype	Control	Case	OR (95%CI)	*p*
rs77941834	Codominant	TT	57	210	1.00	
		TA	23	46	**0.52 (0.29–0.94)**	**0.030**
		AA	4	5	0.33 (0.09–1.31)	0.116
	Dominant	TT	57	210	1.00	
		TA/AA	27	51	**0.49 (0.28–0.86)**	**0.013**
	Recessive	TT/TA	80	256	1.00	
		AA	4	5	0.39 (0.10–1.49)	0.167
	Log-additive				**0.55 (0.34–0.87)**	**0.011**

SNP = single nucleotide polymorphism; OR = odds ratio; 95%CI = 95% confidence interval.

*p* < 0.05 indicates statistical significance.

Bold values indicate a significant difference.

### Stratification analysis of age and gender

Multiple inheritance model analysis showed that age and sex significantly affected the association between *COL6A4P2* SNPs and LC risk. We found that rs34445363 was associated with a higher incidence of LC in people aged ≤ 61 years with the AA genotype in the codominant model (adjusted by gender, AA vs. GG, OR = 2.62, 95%CI: 1.00–6.85, *p* = 0.049) and in the log-additive model (adjusted by gender, OR = 1.42, 95%CI: 1.03–1.95, *p* = 0.033); rs61733464 was associated with a decreased LC risk under the dominant model (adjusted by gender, GA/AA vs. GG, OR = 0.68, 95%CI: 0.46–0.99; *p* = 0.048) and log-additive model (adjusted by gender, OR = 0.72, 95%CI: 0.52–0.99, *p* = 0.048) in people aged ≤ 61 years (Table 5).

**Table 5:** Distribution of COL6A4P2 polymorphisms in populations of different ages and genders and its association with risk of lung cancer.

SNP ID	Model	Genotype	Age > 61				Age ≤ 61			
			Control	Case	OR (95%CI)	*p*	Control	Case	OR (95%CI)	*p*
rs34445363	Codominant	GG	179	152	1.00		150	161	1.00	
		GA	82	86	1.24 (0.85–1.81)	0.254	64	87	1.29 (0.87–1.92)	0.210
		AA	9	6	0.76 (0.26–2.19)	0.606	6	18	**2.62 (1.00–6.85)**	**0.049**
	Dominant	GG	179	152	1.00		150	161	1.00	
		GA/AA	91	92	1.20 (0.83–1.72)	0.340	70	105	1.41 (0.96–2.06)	0.079
	Recessive	GG/GA	261	238	1.00		214	248	1.00	
		AA	9	6	0.70 (0.24–2.02)	0.513	6	18	2.41 (0.93–6.24)	0.070
	Log-additive	--	--	--	1.11 (0.81–1.53)	0.524	--	--	**1.42 (1.03–1.95)**	**0.033**
rs61733464	Codominant	GG	174	159	1.00		136	181	1.00	
		GA	81	74	0.98 (0.67–1.44)	0.923	77	76	0.70 (0.47–1.03)	0.073
		AA	15	11	0.82 (0.36–1.86)	0.636	11	9	0.58 (0.23–1.46)	0.249
	Dominant	GG	174	159	1.00		136	181	1.00	
		GA/AA	96	85	0.96 (0.66–1.38)	0.812	88	85	**0.68 (0.46–0.99)**	**0.048**
	Recessive	GG/GA	255	233	1.00		213	257	1.00	
		AA	15	11	0.83 (0.37–1.86)	0.642	11	9	0.66 (0.26–1.63)	0.365
	Log-additive	--	--	--	0.95 (0.70–1.28)	0.713	--	--	**0.72 (0.52–0.99)**	**0.048**
SNP ID	Model	Genotype	Male				Female			
			Control	Case	OR (95%CI)	*p*	Control	Case	OR (95%CI)	*p*
rs34445363	Codominant	GG	225	220	1.00		104	92	1.00	
		GA	110	118	1.10 (0.80–1.52)	0.547	36	55	**1.73 (1.04–2.86)**	**0.034**
		AA	11	17	1.47 (0.67–3.24)	0.334	4	7	1.98 (0.56–6.98)	0.289
	Dominant	GG	225	220	1.00		104	92	1.00	
		GA/AA	121	135	1.14 (0.84–1.55)	0.411	40	62	**1.75 (1.08–2.85)**	**0.024**
	Recessive	GG/GA	335	338	1.00		140	147	1.00	
		AA	11	17	1.43 (0.65–3.11)	0.372	4	7	1.67 (0.18–5.82)	0.423
	Log-additive	--	--	--	1.15 (0.88–1.49)	0.314	--	--	**1.60 (1.05–2.44)**	**0.028**
rs77941834	Codominant	TT	279	284	1.00		124	112	1.00	
		TA	63	61	0.94 (0.64–1.39)	0.763	23	39	**1.88 (1.06–3.34)**	**0.032**
		AA	4	10	2.42 (0.75–7.84)	0.141	1	2	2.21 (0.20–24.76)	0.519
	Dominant	TT	279	284	1.00		124	112	1.00	
		TA/AA	67	71	1.03 (0.71–1.50)	0.878	24	41	**1.89 (1.07–3.33)**	**0.027**
	Recessive	TT/TA	342	345	1.00		147	151	1.00	
		AA	4	10	2.44 (0.76–7.91)	0.136	1	2	1.94 (0.17–21.66)	0.590
	Log-additive	--	--	--	1.11 (0.80–1.53)	0.547	--	--	**1.81 (1.06–3.08)**	**0.030**

SNP = single nucleotide polymorphism; OR = odds ratio; 95%CI = 95% confidence interval.

*p* < 0.05 indicates statistical significance.

Bold values indicate a significant difference.

In addition, we found that the sex significantly affected the association between SNPs of the *COL6A4P2* gene and LC risk (Table 5). The mutation of *COL6A4P2* rs34445363 in females could significantly increase the LC risk with the GA genotype under the codominant model (adjusted by age, GA vs. GG, OR = 1.73, 95% CI: 1.04–2.86, *p* = 0.034), dominant model (adjusted by age, GA/AA vs. GG, OR = 1.75, 95% CI: 1.08–2.85, *p* = 0.024) and log-additive model (adjusted by age, OR = 1.60, 95% CI: 1.05–2.44, *p* = 0.028); Women with rs77941834 mutation have a higher incidence of LC with the TA genotype under the codominant model (adjusted by age, TA vs. TT, OR = 1.88, 95% CI: 1.06–3.34, *p* = 0.032), in dominant model (adjusted by age, TA/AA vs. TT, OR = 1.89, 95% CI: 1.07–3.33, *p* = 0.027) and log-additive model (adjusted by age, OR = 1.81, 95% CI: 1.06–3.08, *p* = 0.030).

### FPRP analysis

The results of FPRP analysis showed that (S2 Table): the association between COL6A4P2 rs34445363 and LC in people aged ≤ 61 (p = 0.049) was not noteworthy at the prior probability level of 0.25 and FPRP threshold of 0.2 (FPRP = 0.338). The FPRP of the remaining significant results were all less than 0.2, which means that these positive results were noteworthy.

### Association of COL6A4P2 haplotypes with the risk of LC

**Fig 1:**
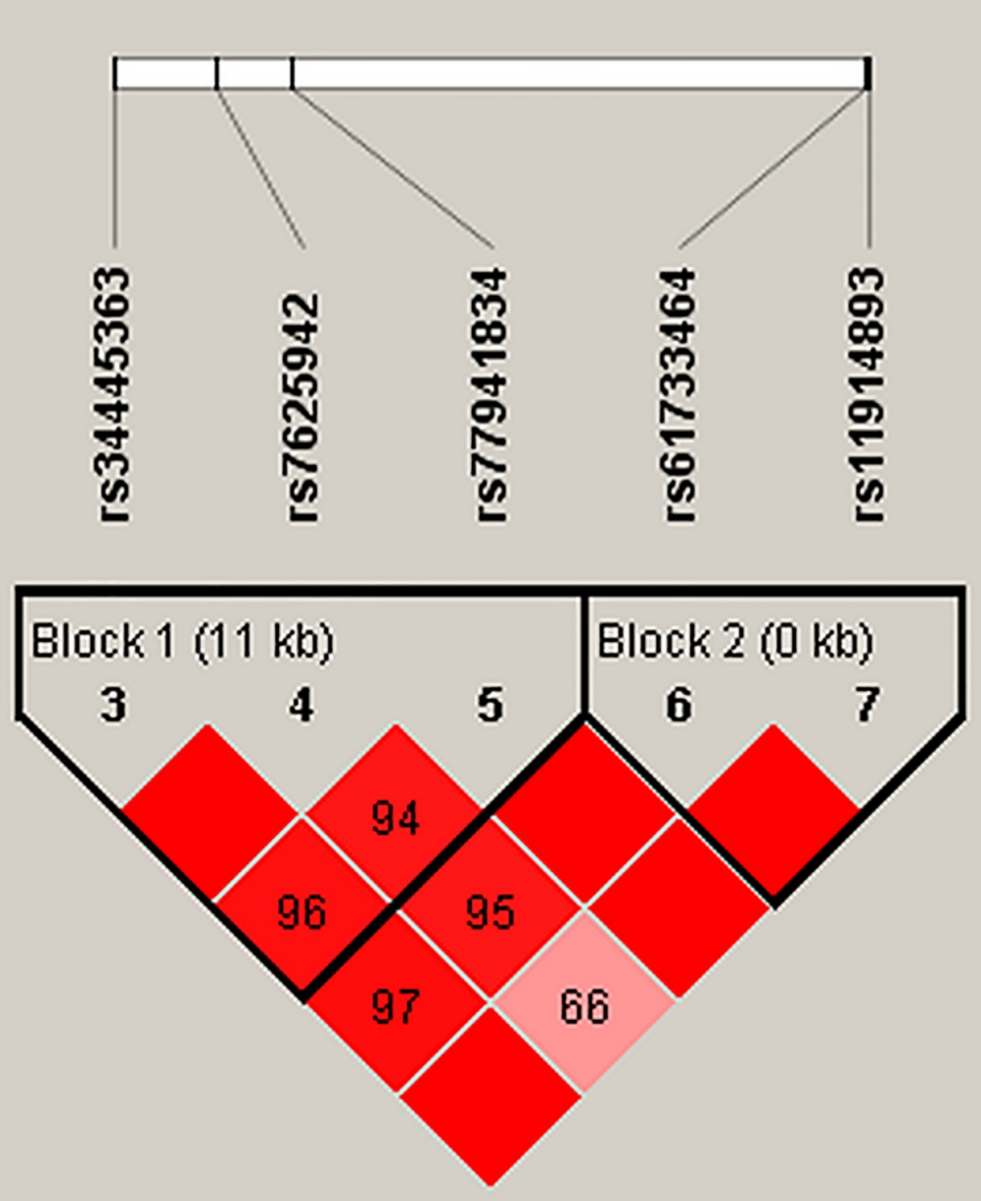
Haplotype block map for SNPs of the *COL6A4P2* gene. Linkage disequilibrium plots containing 5 SNPs from *COL6A4P2*. Red squares display statistically significant associations between a pair of SNPs, as measured by D’; darker shades of red indicate a higher D’.

SNPs in the current study were in linkage disequilibrium for the study population (Fig 1). Unfortunately, there was no statistically significant difference among the *COL6A4P2* haplotype frequencies in the cases and controls (S3 Table).

### Expression of COL6A4P2 and SNPs

**Fig 2:**
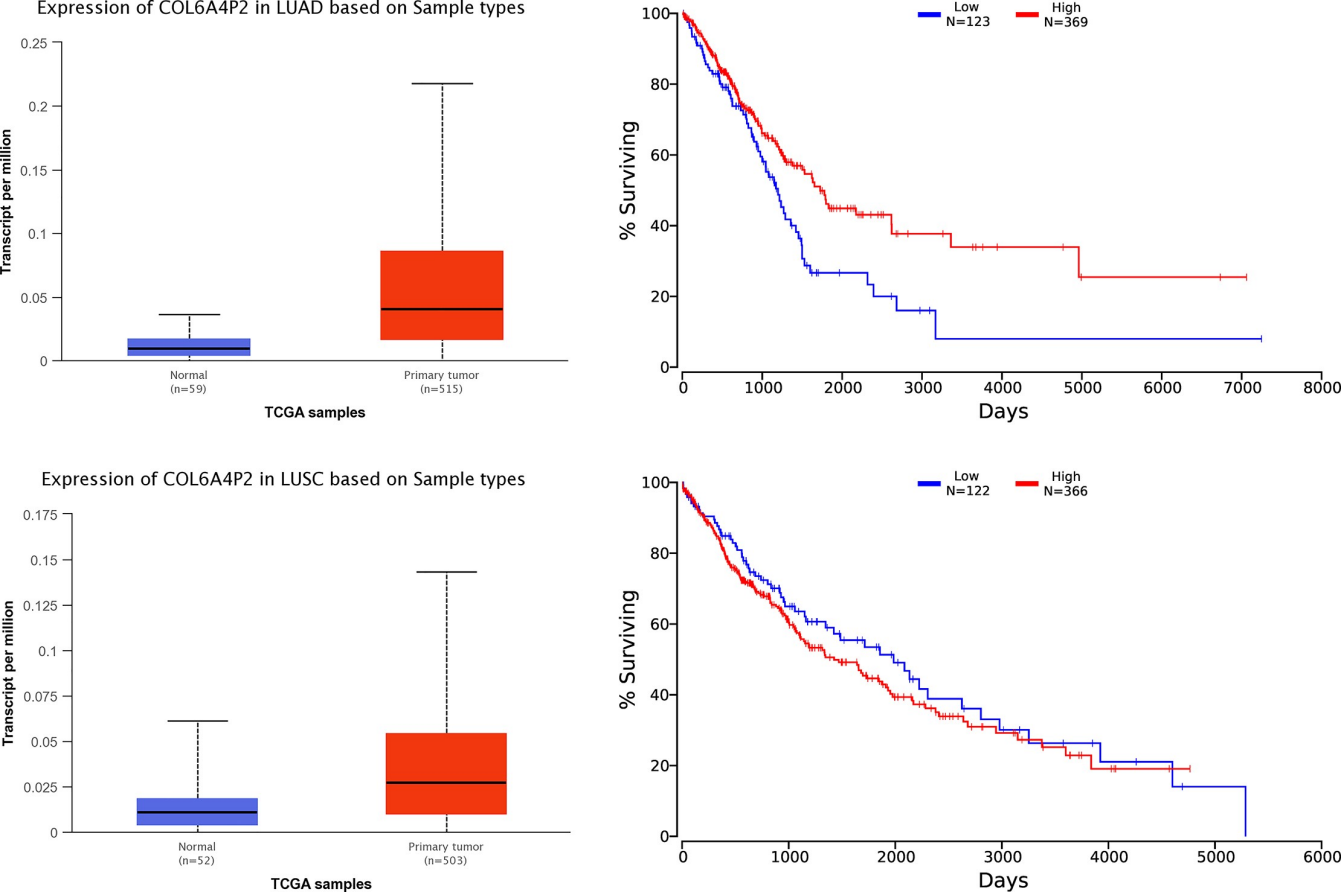
Prediction of the expression and prognosis of *COL6A4P2* gene in LUAD and LUSC. (A) Expression of *COL6A4P2* in LUAD and normal tissues (*p* = 1.62 × 10^−12^). (B) Effect of *COL6A4P2* gene expression on survival rate (*p* = 4.25 × 10^−3^). (C) Expression of *COL6A4P2* in LUSC and normal tissues (*p* = 2.44 × 10^−7^). (D) Effect of *COL6A4P2* gene expression on survival rate (*p* = 3.00 × 10^−1^).

**Fig 3:**
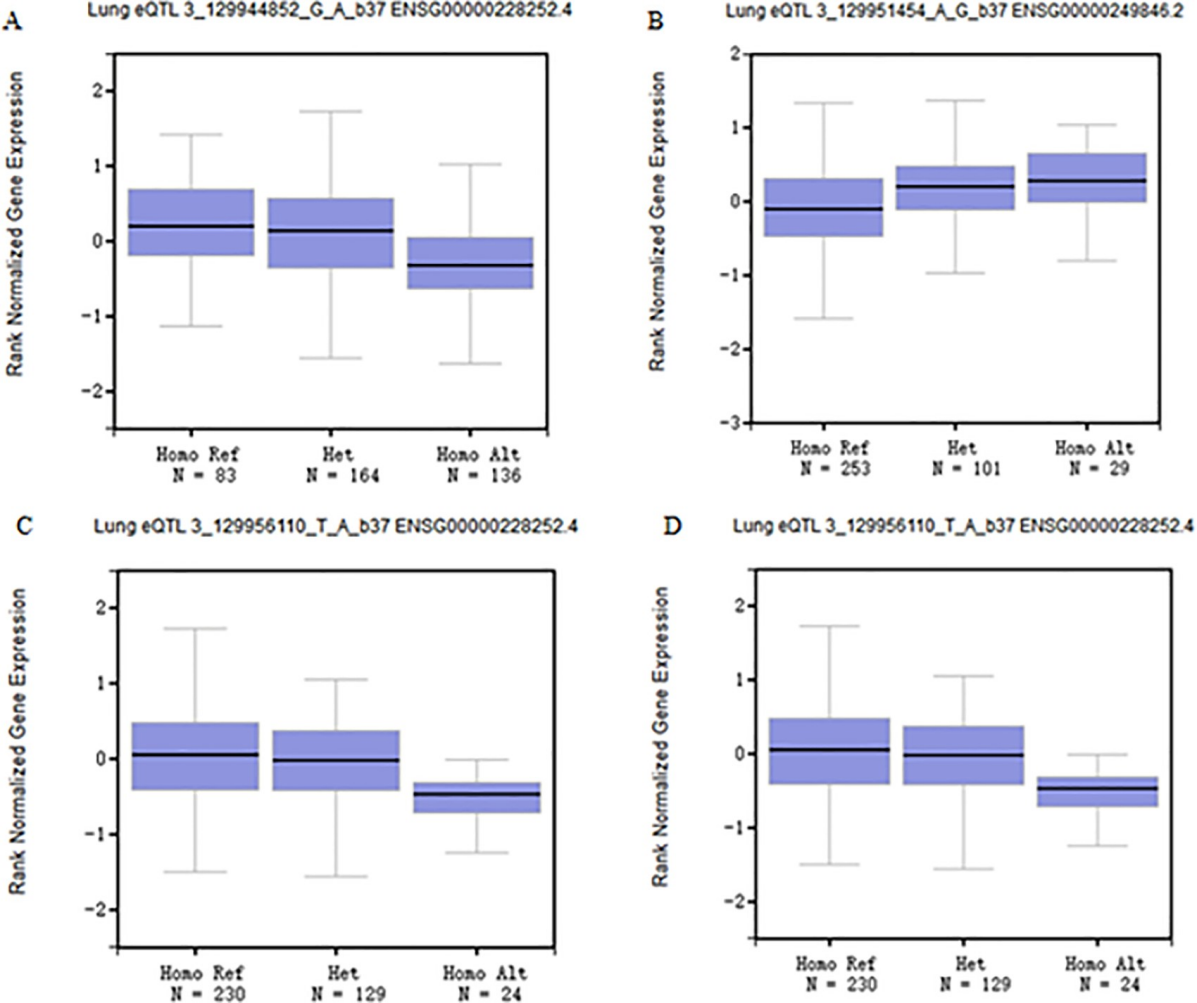
Expression of *COL6A4P2* SNPs in lung tissues. (A) Expression of rs34445363 genotype in the lung (*p* = 5.80 × 10^−14^); (B) Expression of rs7625942 genotype in the lung (*p* = 8.90 × 10^−8^); (C) Expression of rs77941834 genotype in the lung (*p* = 1.60 × 10^−5^); (D) Expression of rs61733464 genotype in the lung (*p* = 1.00 × 10^−9^).

Database analysis showed that compared with healthy subjects, expression of the *COL6A4P2* gene was significantly higher in LUAD (*p* = 1.62 × 10^−12^), and expression of the *COL6A4P2* gene was significantly higher in LUSC (*p* = 2.44 × 10^−7^, Fig 2A and 2C). OncoLnc database analysis showed that expression of the *COL6A4P2* gene was significantly correlated with the survival rate in LUAD patients (Fig 2B, *p* = 4.25 × 10^−3^). However, the expression of the *COL6A4P2* gene had no significant effect on the prognosis of LUSC (*p* = 3.00 × 10^−1^, Fig 2D). Furthermore, the GTEx database prediction results showed that four SNPs (rs34445363, *p* = 5.80 × 10^−14^; rs7625942, *p* = 8.90 × 10^−8^; rs77941834, *p* = 1.60 × 10^−5^; rs61733464, *p* = 1.00 × 10^−9^) on the *COL6A4P2* gene were significantly expressed in normal lung tissues (Fig 3).

## Discussion

In this study we analyzed the association of *COL6A4P2* gene polymorphisms with susceptibility to LC. We identified that rs34445363 in *COL6A4P2* was associated with an increased risk of LC. Our results also suggested that rs34445363 site mutations increase the risk of LUAD, while the mutation of rs61733464 significantly decreased the LUAD risk. These results suggest an association between genetic polymorphisms of *COL6A4P2* and susceptibility to LC.

Numerous studies have shown that collagen levels play an essential role in the development of LC [20, 21]. Naveen *et al*. [22] identified collagen VI as a potential biomarker for the early diagnosis of LC by proteomic analysis, suggesting that LC is associated with collagen-encoding genes. The *COL6A4P2* gene is a pseudogene formed by the chromosomal break of the collagen-encoding gene *COL6A4* [13, 23]; therefore, we speculate that the *COL6A4P2* gene may be associated with LC. Our results suggest that the rs34445363 mutation in the *COL6A4P2* gene significantly increases the risk of LC, validating our conjecture, and is consistent with previous studies.

Our results also found that the association between *COL6A4P2* gene polymorphism and LC risk was influenced by gender and age. A retrospective analysis by Oh *et al*. [24] assessed the crucial effects of sex and age in the development of LC. Aareleid *et al*. [25] revealed that LC has different incidence rates in different genders and ages. These studies are consistent with our results and enhance the credibility of our findings.

Furthermore, we predicted the differential expression of *COL6A4P2* in normal lung tissues and LC tissues using a database. Voiles *et al*. [12] found that collagen VI protein levels increased in tumor lung tissue and speculated that the expression of the *COL6A4P2* gene in tumor lung tissue is variable. This is consistent with our predictions. Fagerberg *et al*. [26] found that the *COL6A4P2* gene is specifically expressed in human lung tissue by genome-wide integration analysis of transcriptomics and antibody proteomics. These findings suggest the important research significance of the *COL6A4P2* gene in the development of LC, prompting that the *COL6A4P2* gene deserves further study.

## Conclusion

In conclusion, the present study is the first to investigate the association between *COL6A4P2* and LC. Our findings indicated that *COL6A4P2* gene polymorphism is associated with LC risk in the Chinese Han population. However, it is necessary to conduct further studies in other races and larger sample sizes to confirm our results. Our study provides a basic reference for individualized LC prevention.

## Supporting information

S1 Table Association between *COL6A4P2* polymorphism and lymph node metastasis status in patients with lung cancer. (DOCX)

S2 Table FPRP and statistical power values of the association analysis results in the subgroup analysis. (DOCX)

S3 Table Haplotype frequencies of *COL6A4P2* polymorphisms and their association with the risk of lung cancer. (DOCX)

S4 Table Genotyping results of all participants. (XLSX)

S1 File (DOCX)
