# Supplementary material for: Impact of COL6A4P2 gene polymorphisms on the risk of lung cancer: A case-control study
Source: PLoS One. 2021 May 21;16(5):e0252082. doi: 10.1371/journal.pone.0252082 (PMC8139505; doi:10.1371/journal.pone.0252082)
Supplement: S1 Table — (DOCX) [file pone.0252082.s001.docx]

**S1 Table.** Association between *COL6A4P2* polymorphism and lymph node metastasis status in patients with lung cancer.

| SNP ID | Model | Genotype | Negative | Positive | OR (95%CI) | *p* |
| --- | --- | --- | --- | --- | --- | --- |
| rs34445363 | Codominant | GG | 50 | 130 | 1.00 |  |
|  |  | GA | 29 | 75 | 0.98(0.57-1.69) | 0.949 |
|  |  | AA | 5 | 10 | 0.74(0.24-2.30) | 0.601 |
|  | Dominant | GG | 50 | 130 | 1.00 |  |
|  |  | GA/AA | 34 | 85 | 0.95(0.56-1.59) | 0.838 |
|  | Recessive | GG/GA | 79 | 205 | 1.00 |  |
|  |  | AA | 5 | 10 | 0.74(0.24-2.28) | 0.603 |
|  | Log-additive | -- | -- | -- | 0.92(0.61-1.42) | 0.717 |
| rs7625942 | Codominant | AA | 55 | 131 | 1.00 |  |
|  |  | AG | 25 | 73 | 1.20(0.69-2.10) | 0.517 |
|  |  | GG | 4 | 11 | 1.05（0.31-3.48） | 0.943 |
|  | Dominant | AA | 55 | 131 | 1.00 |  |
|  |  | AG/GG | 29 | 84 | 1.18(0.69-2.01) | 0.540 |
|  | Recessive | AA/AG | 80 | 204 | 1.00 |  |
|  |  | GG | 4 | 11 | 0.98(0.30-3.22) | 0.977 |
|  | Log-additive | -- | -- | -- | 1.12(0.72-1.74) | 0.618 |
| rs77941834 | Codominant | TT | 64 | 170 | 1.00 |  |
|  |  | TA | 17 | 41 | 0.86(0.46-1.64) | 0.660 |
|  |  | AA | 3 | 4 | 0.53(0.12-2.46) | 0.421 |
|  | Dominant | TT | 64 | 170 | 1.00 |  |
|  |  | TA/AA | 20 | 45 | 0.82(0.45-1.50) | 0.511 |
|  | Recessive | TT/TA | 81 | 211 | 1.00 |  |
|  |  | AA | 3 | 4 | 0.55(0.12-2.52) | 0.44 |
|  | Log-additive | -- | -- | -- | 0.81(0.48-1.35) | 0.417 |
| rs61733464 | Codominant | GG | 60 | 141 | 1.00 |  |
|  |  | GA | 21 | 66 | 1.31(0.73-2.34) | 0.362 |
|  |  | AA | 3 | 8 | 0.98(0.25-3.92) | 0.982 |
|  | Dominant | GG | 60 | 141 | 1.00 |  |
|  |  | GA/AA | 24 | 74 | 1.27(0.73-2.21) | 0.400 |
|  | Recessive | GG/GA | 81 | 207 | 1.00 |  |
|  |  | AA | 3 | 8 | 0.91(0.23-3.60) | 0.894 |
|  | Log-additive | -- | -- | -- | 1.18(0.73-1.90) | 0.499 |
| rs11914893 | Codominant | AA | 69 | 169 | 1.00 |  |
|  |  | AC | 15 | 43 | 1.21(0.63-2.33) | 0.571 |
|  |  | CC | 0 | 3 |  | 0.999 |
|  | Dominant | AA | 69 | 169 | 1.00 |  |
|  |  | AC/CC | 15 | 46 | 1.30(0.68-2.50) | 0.428 |
|  | Recessive | AA/AC | 84 | 212 | 1.00 |  |
|  |  | CC | 0 | 3 |  | 0.999 |
|  | Log-additive | -- | -- | -- | 1.37(0.74-2.54) | 0.314 |

SNP = single nucleotide polymorphism; OR = odds ratio; 95%CI = 95% confidence interval.

p < 0.05 indicates statistical significance.

Bold values indicate a significant difference.
