# Supplementary material for: Impact of COL6A4P2 gene polymorphisms on the risk of lung cancer: A case-control study
Source: PLoS One. 2021 May 21;16(5):e0252082. doi: 10.1371/journal.pone.0252082 (PMC8139505; doi:10.1371/journal.pone.0252082)
Supplement: S2 Table — (DOCX) [file pone.0252082.s002.docx]

**S2 Table.** The FPRP and statistical power values of the association analysis results in the subgroup analysis.

| SNP ID | Model | Genotype | OR (95%CI) | *p* | Statistical Power ^a^ (%) | Prior probability | | |
| --- | --- | --- | --- | --- | --- | --- | --- | --- |
|  |  |  |  |  |  | 0.25 | 0.1 | 0.01 |
| **Subgroup analysis: tumor stage** | | | | | | | | |
| rs77941834 | Codominant | TA | 0.52 (0.29 - 0.94) | 0.030 | 55.2 | 0.142 ^b^ | 0.332 | 0.845 |
|  | Dominant | TA/AA | 0.49 (0.28 - 0.86) | 0.013 | 47.2 | 0.076 ^b^ | 0.198 ^b^ | 0.731 |
|  | Log-additive | - | 0.55 (0.34 - 0.87) | 0.011 | 65.8 | 0.046 ^b^ | 0.127 ^b^ | 0.615 |
| **Subgroup analysis: age (age ≤ 61)** | | | | | | | | |
| rs34445363 | Codominant | AA | 2.62 (1.00 - 6.85) | 0.049 | 29.1 | 0.338 | 0.605 | 0.944 |
|  | Log-additive | - | 1.42 (1.03 - 1.95) | 0.033 | 98.3 | 0.085 ^b^ | 0.217 | 0.753 |
| rs61733464 | Dominant | GA/AA | 0.68 (0.46 - 0.99) | 0.048 | 94.6 | 0.123 ^b^ | 0.296 | 0.822 |
|  | Log-additive | - | 0.72 (0.52 - 0.99) | 0.048 | 98.8 | 0.116 ^b^ | 0.282 | 0.812 |
| **Subgroup analysis: gender (female)** | | | | | | | | |
| rs34445363 | Codominant | GA | 1.73 (1.04 - 2.86) | 0.034 | 71.4 | 0.120 ^b^ | 0.291 | 0.819 |
|  | Dominant | GA/AA | 1.75 (1.08 - 2.85) | 0.024 | 70.4 | 0.095 ^b^ | 0.239 | 0.775 |
|  | Log-additive | - | 1.60 (1.05 - 2.44) | 0.028 | 85.0 | 0.093 ^b^ | 0.235 | 0.772 |
| rs77941834 | Codominant | TA | 1.88 (1.06 - 3.34) | 0.032 | 58.4 | 0.139 ^b^ | 0.326 | 0.842 |
|  | Dominant | TA/AA | 1.89 (1.07 - 3.33) | 0.027 | 57.8 | 0.125 ^b^ | 0.301 | 0.826 |
|  | Log-additive | - | 1.81 (1.06 - 3.08) | 0.030 | 64.4 | 0.118 ^b^ | 0.286 | 0.815 |

FPRP: false-positive report probability.

^a^ Statistical power ^a^ was calculated using the number of observations in the subgroup and the OR and p values in this table.

^b^ The level of false-positive report probability threshold was set at 0.2, and noteworthy findings are presented.
