# Supplementary material for: Impact of COL6A4P2 gene polymorphisms on the risk of lung cancer: A case-control study
Source: PLoS One. 2021 May 21;16(5):e0252082. doi: 10.1371/journal.pone.0252082 (PMC8139505; doi:10.1371/journal.pone.0252082)
Supplement: S3 Table — (DOCX) [file pone.0252082.s003.docx]

**S3 Table.** The haplotype frequencies of *COL6A4P2* polymorphisms and their association with the risk of lung cancer.

| Haplotype | Freq.(case) | Freq.(control) | OR (95%CI) | *p* |
| --- | --- | --- | --- | --- |
| GAT | 0.558 | 0.593 | 1.00 |  |
| AAA | 0.883 | 0.903 | 0.81(0.61-1.08) | 0.156 |
| GGT | 0.779 | 0.772 | 1.04(0.84-1.28) | 0.722 |
| AAT | 0.901 | 0.918 | 0.81(0.60-1.11) | 0.187 |
| GA | 0.706 | 0.672 | 1.00 |  |
| GC | 0.892 | 0.885 | 1.08(0.82-1.43) | 0.569 |
| AA | 0.814 | 0.787 | 1.18(0.95-1.46) | 0.139 |

OR = odds ratio; 95%CI = 95% confidence interval.

p < 0.05 indicates statistical significance.

Bold values indicate a significant difference.
