# Supplementary material for: Impact of COL6A4P2 gene polymorphisms on the risk of lung cancer: A case-control study
Source: PLoS One. 2021 May 21;16(5):e0252082. doi: 10.1371/journal.pone.0252082 (PMC8139505; doi:10.1371/journal.pone.0252082)
Supplement: S1 File — (DOCX) [file pone.0252082.s005.docx]

**Impact of *COL6A4P2* gene polymorphisms on the risk of** **lung cancer in a Chinese Han population: a case-control study**

**Short title:  *COL6A4P2* gene polymorphisms and lung cancer in a Chinese Han population**

Xiaodong Dang^1^, Wenhui Zhao^1^, Chen Li^2^, Hua Yang^2^, Dianzhen Li^2^, Shanshan Zhang^2^, Tianbo Jin^3, 4, #^

^1^ Department of Anesthesiology, Shaanxi Provincial Cancer Hospital, Shaanxi Xi’an 710061, China

^2^ Xi'an 21st Century Biological Sicence and Technology Co., Ltd, Shaanxi Xi’an 712000, China

^3^ Key Laboratory of Molecular Mechanism and Intervention Research for Plateau Diseases of Tibet Autonomous Region, School of Medicine, Xizang Minzu University, Xianyang, Shaanxi 712082, China.

^4^ Key Laboratory of Resource Biology and Biotechnology in Western China (Northwest University), Ministry of Education, School of Life Sciences, Northwest University, Xi’an, Shaanxi 710069, China.

^#^ Corresponding Author:

Tianbo Jin

Tel/Fax: +86-029-88895902

E-mail: tianbo__jin@163.com

Address: # 6 East Wenhui Road, Xianyang 712082, Shaanxi, China

**Declarations**

**Consent to publication**

All the authors agreed to publish the manuscript.

**Availability of data and materials**

All original data used in this study can be found in S4 Table.

**Funding**

The author(s) received no specific funding for this work. Our study had no funding relationship with Xi'an 21st Century Biological Sicence and Technology Co., Ltd, which has an employment relationship with several authors of this study. The specific roles of these authors are articulated in the ‘author contributions’ section.

**Competing interests**

The authors declare that they have no conflict of interest.

**Author contribution**

The work presented here was carried out in collaboration between all authors. Xiaodong Dang carried out the molecular genetic studies and drafted the manuscript. Xiaodong Dang and Wenhui Zhao designed the methods and experiments, performed the statistical analyses and interpreted the results. Chen Li and Hua Yang designed primers and performed DNA extraction and purification. Chen Li, Hua Yang, Dianzhen Li and Shanshan Zhang performed the SNP genotyping experiments. Wenhui Zhao worked on associated data collection and their interpretation. Tianbo Jin conceived of the study, participated in the design and coordination of the study. All authors read and approved the final manuscript.

**Abstract**

Lung cancer (LC) is a malignant tumor that poses the greatest threat to human health and life. Most studies have suggested that the occurrence of LC is associated with environmental and genetic factors. We aimed to explore the association between *COL6A4P2* single nucleotide polymorphisms (SNPs) and CHD risk in the Chinese Southern Han population. Based on the ‘case-control’ experimental design (510 cases and 495 controls), we conducted an association study between five candidate *COL6A4P2* SNPs and the corresponding LC risk. Odds ratio (OR) and 95% confidence intervals (CIs) were calculated by logistic regression to analyze the LC susceptibility under different genetic models. The results showed that *COL6A4P2* rs34445363 was significantly associated with LC risk under allele model (OR = 1.26, 95% CI: 1.01 - 1.58, *p* = 0.038). In addition, rs34445363 was also significantly associated with LC risk in the log-additive model (OR = 1.26, 95% CI: 1.01 - 1.58, *p* = 0.041). The results of subgroup analysis showed that rs34445363 (OR = 1.42, 95% CI: 1.03 - 1.95, p = 0.033) and rs61733464 (OR = 0.72, 95% CI: 0.52 - 0.99, *p* = 0.048) were both significantly associated with LC risk in the log-additive model among participants who were ≤ 61 years old. We also found that the variation of rs34445363 (GA vs. GG, OR = 1.73, 95% CI: 1.04 - 2.86, *p* = 0.034) and rs77941834 (TA vs. TT, OR = 1.88, 95% CI: 1.06 - 3.34, *p* = 0.032) were associated with LC risk in the codominant model among female participants. Our study is the first to find that *COL6A4P2* gene polymorphism is associated with LC risk in the Chinese Han population. Our study provides a basic reference for individualized LC prevention.

**Keywords**: Lung cancer, *COL6A4P2* gene, Polymorphisms, Susceptibility

**Introduction**

Lung cancer (LC) is a malignant tumor with the fastest growth in morbidity and mortality and the greatest threat to human health and life ^1^. According to the Global Cancer Observatory database (http://gco.iarc.fr/) ^2^, there are 2,093,876 new cases of LC worldwide, accounting for 11.6% of all cancers; the number of people who died of LC in 2018 is 1,761,007, accounting for 17.9% of all cancer deaths in 2018. Among them, the incidence and mortality of LC in women were 13.1% and 6.9%, respectively. LC has become the most malignant tumor with the highest incidence and mortality ^3-5^. In China, LC also has a high incidence and mortality, and its morbidity and mortality in men are more than twice that of women ^6^. Most studies have suggested that the occurrence of LC is associated with environmental (smoke, occupational exposure, and air pollution) and genetic factors. ^7, 8^ In particular, genetic factors play an essential role in the occurrence of LC. Li *et al.* ^9^ revealed that LC susceptibility in the Chinese Han population was associated with *HOTAIR* gene mutations. Dimitrakopoulos *et al.* ^10^ believed that *the NF-kB2* gene mutation is significantly associated with LC risk. However, the association between *COL6A4P2* gene polymorphisms and LC susceptibility has not been reported.

*COL6A4P2* (collagen type VI alpha 4 pseudogene 2), also named *COL6A4*, is located on Chr.3q22 in humans. *COL6A4* expresses type VI collagen (COL6), an extracellular matrix protein that plays a vital role in maintaining lung tissue integrity. Chiu *et al.* ^11^ showed by quantitative secretion cleavage that *COL6* is a protein involved in tumor metastasis. Voiles *et al.* ^12^ demonstrated that the expression of *the COL6* protein in LC is upregulated. Thus, we suspect that *COL6A4* may be associated with LC.

It has been reported that *COL6A4* is an unprocessed pseudogene due to the presence of multiple stop codons in the gene sequence ^13^. Many studies have shown that pseudogenes play an essential role in cancer development; Cheng *et al.* ^14^ have found that pseudogenes affect the occurrence and development of cancer by forming lncRNA-pseudogene-mRNA competitive triples. Lynn *et al.* ^15^ have confirmed that polymorphisms in the *MYLKP1* pseudogene are associated with an increased risk of colon cancer. Wei *et al.* ^16^ have found that the pseudogene *DUXAP10* promotes the invasiveness of LCs. Therefore, we speculated that the *COL6A4P2* gene might play a role in cancer development.

In this study, we first explored the association between the *COL6A4P2* gene and LC risk, revealing the association between *COL6A4P2* gene polymorphism and LC susceptibility in the Chinese Han population.

**Materials and methods**

**Study participants**

Using a case-control design, 510 LC patients (mean age: 60.78 ± 9.96 years) and 495 controls (mean age: 61.94 ± 7.72 years) were enrolled in the study. All patients were recruited from Shaanxi Provincial Cancer Hospital. Patient inclusion criteria were as follows: 1) newly diagnosed LC, 2) histopathological LC diagnosed by an experienced pathologist, 3) no previous radiation therapy or chemical therapy, and 4) no history of cancer or metastatic carcinoma. Patients with asthma, bronchitis, pneumonia, lung abscess, tuberculosis, other lung diseases, autoimmune diseases, trauma, or other tumors were excluded from the study. After that, we investigated and collected information regarding clinical indicators of LC patients, including sex, age, histological classification, tumor stage, and the status of lymph node metastasis.

The controls were healthy volunteers recruited from the Shaanxi Provincial Cancer Hospital during the same period. No medical or family history of cancer or any pulmonary disease was the inclusion criteria for the control group. At the time of recruitment, trained personnel interviewed each subject using a structured questionnaire to obtain information regarding their demographic characteristics.

**Data collection**

This study was approved by the Shaanxi Provincial Cancer Hospital ethics committee and conformed to the ethical principles for medical research involving humans of the World Medical Association Declaration of Helsinki. All participants signed informed consent forms before participating in the study. Subsequently, a sample of approximately 5 mL of venous blood was obtained from each participant and collected into tubes containing ethylenediaminetetraacetic acid for anticoagulation. Genomic DNA was extracted from peripheral blood samples using a whole-blood genomic DNA extraction kit (GOLDMAG, Xi´an, China) according to the manufacturer’s instructions. The purity and concentration of the DNA samples were evaluated using a NanoDrop 2000C system (Thermo Scientific, Waltham, MA, USA). Isolated DNA was stored at −80 °C until analysis.

**SNP genotyping**

Five candidate SNPs in the *COL6A4P2* gene were selected with a minor allele frequency (MAF) > 0.05 from the global population in the 1,000 Genome Projects (http://www.internationalgenome.org/). We then used HaploReg v4.1 (https://pubs.broadinstitute.org/mammals/haploreg/haploreg.php) to predict the possible functions of the SNPs. The primers for amplification and single-base extension were designed using the Assay Design Suite V2.0 (https://agenacx.com/online-tools/). Genotyping of the five SNPs was carried out on the MassARRAY iPLEX (Agena Bioscience, San Diego, CA, USA) platform using matrix-assisted laser desorption ionization-time of flight mass spectrometry ^17^. Genotyping results were generated using Agena Bioscience TYPER software, version 4.0. Genotyping was performed by laboratory personnel in a double-blinded manner.

**Analysis of** **COL6A4P2 and SNPs expression**

Data regarding the expression of *COL6A4P2* in LC were obtained from the UALCAN online database (<http://ualcan.path.uab.edu/analysis.html>), a web server that provides customizable functions. Tumors and normal samples in the UALCAN database were derived from The Cancer Genome Atlas (TCGA) and Genotype-Tissue Expression (GTEx) projects. The effect of *COL6A4P2* gene expression on LC prognosis was predicted using the OncoLnc database (<http://www.oncolnc.org/>). We also predicted the expression of SNPs in the *COL6A4P2* gene in normal lung tissues using the GTEx database (<https://gtexportal.org/home/>).

**Statistical analyses**

An independent sample t test was used to assess differences in the population and clinical characteristics of the study participants. Fisher’s exact tests for HWE were performed by comparing the observed and expected genotype frequencies to calculate the genotype frequencies among the controls. Pearson’s χ^2^ test was used to compare the allelic and genotype frequencies of each SNP between LC patients and controls. Multiple genetic model analyses (codominant, dominant, recessive, and log-additive) were performed using PLINK software (http://zzz.bwh.harvard.edu/plink/ld.shtml) to assess the association between SNPs and LC risk. Furthermore, we calculated stratification factors using sex and age to adjust for possible confounders. Finally, we used Haploview software (version4.2) to construct haplotypes and to estimate the pairwise linkage disequilibrium using the SHEsis software platform (http://analysis.bio-x.cn/myAnalysis.php) was used to estimate the association between haplotype and LC risk. Odds ratios (ORs) and 95% confidence intervals (CIs) were calculated using logistic regression analyses adjusted for sex and age ^18^, with the wild-type allele used as a reference. Statistical analyses were performed using SPSS software (version 21.0, IBM Corporation, Armonk, NY, USA). All *p*-values of statistical tests were two-sided, and *p* < 0.05, which was considered indicative of statistical significance. We also conducted a false-positive report probability (FPRP) analysis to detect whether the significant findings were just chance or noteworthy observations ^19^.

**Results**

**Characteristics of cases and controls**

The basic clinical information of patients with LC and controls is shown in Table 1. Five hundred and ten patients were presented with a different distribution, according to age (age ≤ 61, 266 cases; age > 61, 244 cases), gender (male, 355 cases; female, 155 cases), pathological type (lung squamous cell carcinoma [LUSC], 120 patients; lung adenocarcinoma [LUAD], 188 patients), tumor stage (Ⅰ-Ⅱ, 84 cases; Ⅲ-Ⅳ, 261 cases), and lymph node metastasis (LNM) status (positive, 215 cases; negative, 84 cases).

Table 1 The comparison of basic characteristics between cases and controls

| Characteristics | | Case (n=510) | Control (n=495) |
| --- | --- | --- | --- |
| Age | ≤ 61 | 266 | 224 |
|  | > 61 | 244 | 271 |
|  | Mean ± SD | 60.78 ± 9.96 | 61.94 ± 7.72 |
| Gender | Male | 355 | 346 |
|  | Female | 155 | 149 |
| Pathological type | LUSC | 120 |  |
|  | LUAD | 188 |  |
|  | Unknown | 202 |  |
| Tumor stage | Ⅰ-Ⅱ | 84 |  |
|  | Ⅲ-Ⅳ | 261 |  |
|  | Unknown |  |  |
| LNM | Positive | 215 |  |
|  | Negative | 84 |  |
|  | Unknown |  |  |

LUSC = lung squamous cell carcinoma; LUAD = lung adenocarcinoma; LNM = lymph node metastasis

Basic information and allele frequencies of *COL6A4P2* gene polymorphisms are presented in Table 2. The genotype distribution of all SNPs in the control subjects met the HWE (*p* > 0.05). HaploReg function annotation results revealed that SNPs associated with LC risk were successfully predicted to have biological functions. The association between *COL6A4P2* polymorphisms and LC risk under the allele model is shown in Table 2, and the results showed that rs34445363 was associated with an increased LC risk (OR = 1.26, 95%CI: 1.01 - 1.58, *p* = 0.038), and there were no differences between the other four SNPs (rs7625942, rs77941834, rs61733464, and rs11914893) in the *COL6A4P2* gene and LC risk (*p* > 0.05).

Table 2 Basic Information about SNPs in COL6A4P2 and association with risk of lung cancer in allele model

| Gene | SNP ID | Chr. | Alleles(A/B) | Frequency (MAF) | | *p*-value for HWE | OR (95% CI) | *p* | Function |
| --- | --- | --- | --- | --- | --- | --- | --- | --- | --- |
|  |  |  |  | Case | Control |  |  |  |  |
| COL6A4P2 | rs34445363 | 3q22.1 | A/G | 0.217 | 0.180 | 0.879 | **1.26 (1.01 - 1.58)** | **0.038** | Selected eQTL hits |
| COL6A4P2 | rs7625942 | 3q22.1 | A/G | 0.223 | 0.225 | 0.608 | 0.98 (0.80 - 1.21) | 0.915 | Motifs changed, Selected eQTL hits |
| COL6A4P2 | rs77941834 | 3q22.1 | A/T | 0.122 | 0.097 | 0.798 | 1.29 (0.97 - 1.71) | 0.086 | Motifs changed, Selected eQTL hits |
| COL6A4P2 | rs61733464 | 3q22.1 | A/G | 0.186 | 0.213 | 0.346 | 0.85 (0.68 - 1.06) | 0.146 | DNAse, Motifs changed, Selected eQTL hits |
| COL6A4P2 | rs11914893 | 3q22.1 | A/C | 0.108 | 0.115 | 0.825 | 0.93 (0.70 - 1.23) | 0.620 | Motifs changed, GRASP QTL hits |

SNP = single nucleotide polymorphism; Chr. = chromosome; A/B = minor/major, MAF = minor allele frequency; HWE = Hardy Weinberg equilibrium.

*p* < 0.05 indicates statistical significance.

Bold values indicate a significant difference.

**Association between the *COL6A4P2* gene and the risk of LC**

Genetic models (codominant, dominant, recessive, and log-additive) and genotype frequencies were used to identify any associations between the SNPs and the risk of LC. The results showed that rs34445363 in the *COL6A4P2* gene significantly increased the risk of LC in the log-additive model (adjusted for age and sex, OR = 1.26, 95%CI: 1.01. 1.58, *p* = 0.041, Table 3), and no significant difference was found for the other SNPs between cases and controls (all *p* > 0.05).

Table 3 Distribution of genotypes of COL6A4P2 polymorphism depicting their association with lung cancer risk and its histological subtypes

| SNP ID | Model | Genotype | Control | LC | | | LSCC | | | LUAD | | |
| --- | --- | --- | --- | --- | --- | --- | --- | --- | --- | --- | --- | --- |
|  |  |  |  | Case | OR (95%CI) | *p* | Case | OR (95%CI) | *p* | Case | OR (95%CI) | *p* |
| rs34445363 | Codominant | GG | 329 | 313 | 1.00 |  | 72 | 1.00 |  | 112 | 1.00 |  |
|  |  | GA | 146 | 173 | 1.25(0.10-1.64) | 0.102 | 43 | 1.27(0.82-1.96) | 0.278 | 66 | 1.39(0.96-2.00) | 0.082 |
|  |  | AA | 15 | 24 | 1.63(0.84-3.17) | 0.151 | 5 | 1.52(0.52-4.46) | 0.442 | 10 | 1.88(0.81-4.36) | 0.144 |
|  | Dominant | GG | 329 | 313 | 1.00 |  | 72 | 1.00 |  | 112 | 1.00 |  |
|  |  | GA/AA | 161 | 197 | 1.29(0.99-1.67) | 0.056 | 48 | 1.29(0.85-1.97) | 0.229 | 76 | **1.43(1.01-2.04)** | **0.046** |
|  | Recessive | GG/GA | 475 | 486 | 1.00 |  | 115 | 1.00 |  | 178 | 1.00 |  |
|  |  | AA | 15 | 24 | 1.51(0.78-2.92) | 0.220 | 5 | 1.40(0.48-4.06) | 0.533 | 10 | 1.68(0.73-3.87) | 0.223 |
|  | Log-additive | -- | -- | -- | **1.26(1.01-1.58)** | **0.041** | -- | 1.26(0.88-1.80) | 0.212 | -- | **1.38(1.02-1.86)** | **0.034** |
| rs61733464 | Codominant | GG | 310 | 340 | 1.00 |  | 82 | 1.00 |  | 133 | 1.00 |  |
|  |  | GA | 158 | 150 | 0.86(0.66-1.13) | 0.278 | 33 | 0.79(0.50-1.24) | 0.299 | 46 | **0.65(0.44-0.96)** | **0.031** |
|  |  | AA | 26 | 20 | 0.70(0.38-1.28) | 0.246 | 5 | 0.75(0.27-2.07) | 0.581 | 9 | 0.76(0.34-1.69) | 0.504 |
|  | Dominant | GG | 310 | 340 | 1.00 |  | 82 | 1.00 |  | 133 | 1.00 |  |
|  |  | GA/AA | 184 | 170 | 0.84(0.65-1.09) | 0.181 | 38 | 0.78(0.51-1.21) | 0.265 | 55 | **0.66(0.46-0.96)** | **0.031** |
|  | Recessive | GG/GA | 468 | 490 | 1.00 |  | 115 | 1.00 |  | 179 | 1.00 |  |
|  |  | AA | 26 | 20 | 0.73(0.40-1.34) | 0.310 | 5 | 0.81(0.30-2.21) | 0.683 | 9 | 0.87(0.39-1.91) | 0.724 |
|  | Log-additive | -- | -- | -- | 0.85(0.68-1.05) | 0.139 | -- | 0.82(0.57-1.18) | 0.285 | -- | 0.74(0.55-1.01) | 0.059 |

SNP = single nucleotide polymorphism; LC = lung cancer; LUAD = lung adenocarcinoma; LSCC = lung squamous cell carcinoma; OR = odds ratio; 95%CI = 95% confidence interval.

*p* < 0.05 indicates statistical significance.

Bold values indicate a significant difference.

Furthermore, we identified by pathological analysis that rs34445363 locus variation significantly increased the risk of LUAD in the dominant model (adjusted by age and gender, GA/AA vs. GG, OR = 1.43, 95% CI: 1.01 - 2.04, *p* = 0.046) and log-additive model (adjusted by age and gender, OR = 1.38, 95% CI: 1.02 - 1.86, *p* = 0.034); However, mutations of rs61733464 in the *COL6A4P2* gene have a lower incidence of LUAD with the GA genotype in the codominant model (adjusted by age and gender, GA vs. GG, OR = 0.65, 95% CI: 0.44 - 0.96, *p* = 0.031) and under the dominant model (adjusted by age and gender, GA/AA vs. GG, OR = 0.66, 95% CI: 0.46 - 0.96, *p* = 0.031).

**Association between *COL6A4P2* polymorphism and clinicopathological features**

To evaluate the association of *COL6A4P2* SNPs with various clinicopathological features, we segregated patients according to the clinical stage (I–II vs. III–IV) and LNM status (positive vs. negative). There was no significant association between LNM status and *COL6A4P2* polymorphism variation (S1 Table). However, for the rs77941834 variant, the codominant model (adjusted by age and gender, TA vs. TT, OR = 0.52, 95% CI: 0.29. 0.94, *p* = 0.030), dominant model (adjusted by age and gender, TA/AA vs. TT, OR = 0.49, 95% CI: 0.28. 0.86, *p* = 0.013), and log-additive model (adjusted by age and gender, OR = 0.55, 95% CI: 0.34. 0.87, *p* = 0.011) significantly decreased the LC risk in patients with III–IV as compared to patients with III tumor stage (Table 4). No statistically significant association was observed for tumor staging and the other four SNPs (rs34445363, rs7625942, rs61733464, and rs11914893).

Table 4 Association between COL6A4P2 polymorphism and tumor staging of lung cancer

| SNP ID | Model | Genotype | Control | Case | OR (95%CI) | *p* |
| --- | --- | --- | --- | --- | --- | --- |
| rs77941834 | Codominant | TT | 57 | 210 | 1.00 |  |
|  |  | TA | 23 | 46 | **0.52 (0.29 - 0.94)** | **0.030** |
|  |  | AA | 4 | 5 | 0.33 (0.09 - 1.31) | 0.116 |
|  | Dominant | TT | 57 | 210 | 1.00 |  |
|  |  | TA/AA | 27 | 51 | **0.49 (0.28 - 0.86)** | **0.013** |
|  | Recessive | TT/TA | 80 | 256 | 1.00 |  |
|  |  | AA | 4 | 5 | 0.39 (0.10 - 1.49) | 0.167 |
|  | Log-additive |  |  |  | **0.55 (0.34 - 0.87)** | **0.011** |

SNP = single nucleotide polymorphism; OR = odds ratio; 95%CI = 95% confidence interval.

*p* < 0.05 indicates statistical significance.

Bold values indicate a significant difference.

**Stratification analysis of age and gender**

Multiple inheritance model analysis showed that age and sex significantly affected the association between *COL6A4P2* SNPs and LC risk. We found that rs34445363 was associated with a higher incidence of LC in people aged ≤ 61 years with the AA genotype in the codominant model (adjusted by gender, AA vs. GG, OR = 2.62, 95% CI: 1.00 - 6.85, *p* = 0.049) and in the log-additive model (adjusted by gender, OR = 1.42, 95% CI: 1.03 - 1.95, *p* = 0.033); rs61733464 was associated with a decreased LC risk under the dominant model (adjusted by gender, GA/AA vs. GG, OR = 0.68, 95% CI: 0.46 - 0.99; *p* = 0.048) and log-additive model (adjusted by gender, OR = 0.72, 95% CI: 0.52 - 0.99; *p* = 0.048) in people aged ≤ 61 years (Table 5).

Table 5 Distribution of COL6A4P2 polymorphisms in populations of different ages and genders and its association with risk of lung cancer

| SNP ID | Model | | Genotype | | Age > 61 | | | | | | | Age ≤ 61 | | | | | | | |  |
| --- | --- | --- | --- | --- | --- | --- | --- | --- | --- | --- | --- | --- | --- | --- | --- | --- | --- | --- | --- | --- |
|  |  |  |  |  | Control | | Case | OR (95%CI) | | *p* | | Control | | Case | | OR (95%CI) | | *p* | |  |
| rs34445363 | Codominant | | GG | | 179 | | 152 | 1.00 | |  | | 150 | | 161 | | 1.00 | |  | |  |
|  |  | | GA | | 82 | | 86 | 1.24 (0.85 - 1.81) | | 0.254 | | 64 | | 87 | | 1.29 (0.87 - 1.92) | | 0.210 | |  |
|  |  | | AA | | 9 | | 6 | 0.76 (0.26 - 2.19) | | 0.606 | | 6 | | 18 | | **2.62 (1.00 - 6.85)** | | **0.049** | |  |
|  | Dominant | | GG | | 179 | | 152 | 1.00 | |  | | 150 | | 161 | | 1.00 | |  | |  |
|  |  | | GA/AA | | 91 | | 92 | 1.20 (0.83 - 1.72) | | 0.340 | | 70 | | 105 | | 1.41 (0.96 - 2.06) | | 0.079 | |  |
|  | Recessive | | GG/GA | | 261 | | 238 | 1.00 | |  | | 214 | | 248 | | 1.00 | |  | |  |
|  |  | | AA | | 9 | | 6 | 0.70 (0.24 - 2.02) | | 0.513 | | 6 | | 18 | | 2.41 (0.93 - 6.24) | | 0.070 | |  |
|  | Log-additive | | -- | | -- | | -- | 1.11 (0.81 - 1.53) | | 0.524 | | -- | | -- | | **1.42 (1.03 - 1.95)** | | **0.033** | |  |
| rs61733464 | Codominant | | GG | | 174 | | 159 | 1.00 | |  | | 136 | | 181 | | 1.00 | |  | |  |
|  |  | | GA | | 81 | | 74 | 0.98 (0.67 - 1.44) | | 0.923 | | 77 | | 76 | | 0.70 (0.47 - 1.03) | | 0.073 | |  |
|  |  | | AA | | 15 | | 11 | 0.82 (0.36 - 1.86) | | 0.636 | | 11 | | 9 | | 0.58 (0.23 - 1.46) | | 0.249 | |  |
|  | Dominant | | GG | | 174 | | 159 | 1.00 | |  | | 136 | | 181 | | 1.00 | |  | |  |
|  |  | | GA/AA | | 96 | | 85 | 0.96 (0.66 - 1.38) | | 0.812 | | 88 | | 85 | | **0.68 (0.46 - 0.99)** | | **0.048** | |  |
|  | Recessive | | GG/GA | | 255 | | 233 | 1.00 | |  | | 213 | | 257 | | 1.00 | |  | |  |
|  |  | | AA | | 15 | | 11 | 0.83 (0.37 - 1.86) | | 0.642 | | 11 | | 9 | | 0.66 (0.26 - 1.63) | | 0.365 | |  |
|  | Log-additive | | -- | | -- | | -- | 0.95 (0.70 - 1.28) | | 0.713 | | -- | | -- | | **0.72 (0.52 - 0.99)** | | **0.048** | |  |
| SNP ID | Model | Genotype | | Male | | | | | | | | | Female | | | | | | | |
|  |  |  |  | Control | | Case | | | OR (95%CI) | | *p* | | Control | | Case | | OR (95%CI) | | *p* | |
| rs34445363 | Codominant | GG | | 225 | | 220 | | | 1.00 | |  | | 104 | | 92 | | 1.00 | |  | |
|  |  | GA | | 110 | | 118 | | | 1.10 (0.80 - 1.52) | | 0.547 | | 36 | | 55 | | **1.73 (1.04 - 2.86)** | | **0.034** | |
|  |  | AA | | 11 | | 17 | | | 1.47 (0.67 - 3.24) | | 0.334 | | 4 | | 7 | | 1.98 (0.56 - 6.98) | | 0.289 | |
|  | Dominant | GG | | 225 | | 220 | | | 1.00 | |  | | 104 | | 92 | | 1.00 | |  | |
|  |  | GA/AA | | 121 | | 135 | | | 1.14 (0.84 - 1.55) | | 0.411 | | 40 | | 62 | | **1.75 (1.08 - 2.85)** | | **0.024** | |
|  | Recessive | GG/GA | | 335 | | 338 | | | 1.00 | |  | | 140 | | 147 | | 1.00 | |  | |
|  |  | AA | | 11 | | 17 | | | 1.43 (0.65 - 3.11) | | 0.372 | | 4 | | 7 | | 1.67 (0.18 - 5.82) | | 0.423 | |
|  | Log-additive | -- | | -- | | -- | | | 1.15 (0.88 - 1.49) | | 0.314 | | -- | | -- | | **1.60 (1.05 - 2.44)** | | **0.028** | |
| rs77941834 | Codominant | TT | | 279 | | 284 | | | 1.00 | |  | | 124 | | 112 | | 1.00 | |  | |
|  |  | TA | | 63 | | 61 | | | 0.94 (0.64 - 1.39) | | 0.763 | | 23 | | 39 | | **1.88 (1.06 - 3.34)** | | **0.032** | |
|  |  | AA | | 4 | | 10 | | | 2.42 (0.75 - 7.84) | | 0.141 | | 1 | | 2 | | 2.21 (0.20 - 24.76) | | 0.519 | |
|  | Dominant | TT | | 279 | | 284 | | | 1.00 | |  | | 124 | | 112 | | 1.00 | |  | |
|  |  | TA/AA | | 67 | | 71 | | | 1.03 (0.71 - 1.50) | | 0.878 | | 24 | | 41 | | **1.89 (1.07 - 3.33)** | | **0.027** | |
|  | Recessive | TT/TA | | 342 | | 345 | | | 1.00 | |  | | 147 | | 151 | | 1.00 | |  | |
|  |  | AA | | 4 | | 10 | | | 2.44 (0.76 - 7.91) | | 0.136 | | 1 | | 2 | | 1.94 (0.17 - 21.66) | | 0.590 | |
|  | Log-additive | -- | | -- | | -- | | | 1.11 (0.80 - 1.53) | | 0.547 | | -- | | -- | | **1.81 (1.06 - 3.08)** | | **0.030** | |

SNP = single nucleotide polymorphism; OR = odds ratio; 95%CI = 95% confidence interval.

*p* < 0.05 indicates statistical significance.

Bold values indicate a significant difference.

In addition, we found that the gender significantly affected the association between SNPs of the *COL6A4P2* gene and LC risk (Table 5). The mutation of *COL6A4P2* rs34445363 in females could significantly increase the LC risk with the GA genotype under the codominant model (adjusted by age, GA vs. GG, OR = 1.73, 95% CI: 1.04 - 2.86, *p* = 0.034), dominant model (adjusted by age, GA/AA vs. GG, OR = 1.75, 95% CI: 1.08 - 2.85, *p* = 0.024) and log-additive model (adjusted by age, OR = 1.60, 95% CI: 1.05 - 2.44, *p* = 0.028); Women with rs77941834 mutation have a higher incidence of LC with the TA genotype under the codominant model (adjusted by age, TA vs. TT, OR = 1.88, 95% CI: 1.06 - 3.34, *p* = 0.032), in dominant model (adjusted by age, TA/AA vs. TT, OR = 1.89, 95% CI: 1.07 - 3.33, *p* = 0.027) and log-additive model (adjusted by age, OR = 1.81, 95% CI: 1.06 - 3.08, *p* = 0.030).

**FPRP analysis**

The results of FPRP analysis showed that (S2 Table): the association between COL6A4P2 rs34445363 and LC in people aged ≤ 61 (p = 0.049) was not noteworthy at the prior probability level of 0.25 and FPRP threshold of 0.2 (FPRP = 0.338). The FPRP of the remaining significant results were all less than 0.2, which means that these positive results were noteworthy.

**Association of *COL6A4P2* haplotypes with the risk of LC**

SNPs in the current study were in linkage disequilibrium for the study population (Fig 1). Unfortunately, there was no statistically significant difference among the *COL6A4P2* haplotype frequencies in the cases and controls (S3 Table).

**Fig 1. Haplotype block map for SNPs of the *COL6A4P2* gene.** Linkage disequilibrium plots containing 5 SNPs from *COL6A4P2*. Red squares display statistically significant associations between a pair of SNPs, as measured by D’; darker shades of red indicate a higher D’.

**Expression of *COL6A4P2* and SNPs**

Database analysis showed that compared with healthy subjects, expression of the *COL6A4P2* gene was significantly higher in LUAD (*p* = 1.62 × 10^-12^), and expression of the *COL6A4P2* gene was significantly higher in LUSC (*p* = 2.44 × 10^-7^, Fig 2A and 2C). OncoLnc database analysis showed that expression of the *COL6A4P2* gene was significantly correlated with the survival rate in LUAD patients (Fig 2B, *p* = 4.25 × 10^-3^). However, the expression of the *COL6A4P2* gene had no significant effect on the prognosis of LUSC (*p* = 3.00 × 10^-1^, Fig 2D). Furthermore, the GTEx database prediction results showed that four SNPs (rs34445363, *p* = 5.80 × 10^-14^; rs7625942, *p* = 8.90 × 10^-8^; rs77941834, *p* = 1.60 × 10^-5^; rs61733464, *p* = 1.00 × 10^-9^) on the *COL6A4P2* gene were significantly expressed in normal lung tissues (Fig 3).

**Fig 2. Prediction of the expression and prognosis of *COL6A4P2* gene in LUAD and LUSC.** (A) Expression of *COL6A4P2* in LUAD and normal tissues (*p* = 1.62 × 10^-12^). (B) Effect of *COL6A4P2* gene expression on survival rate (*p* = 4.25 × 10^-3^). (C) Expression of *COL6A4P2* in LUSC and normal tissues (*p* = 2.44 × 10^-7^). (D) Effect of *COL6A4P2* gene expression on survival rate (*p* = 3.00 × 10^-1^).

**Fig 3.** **Expression of *COL6A4P2* SNPs in lung tissues.** (A) Expression of rs34445363 genotype in the lung (*p* = 5.80 × 10^-14^). (B) Expression of rs7625942 genotype in the lung (*p* = 8.90 × 10^-8^). (C) Expression of rs77941834 genotype in the lung (*p* = 1.60 × 10^-5^); (D) Expression of rs61733464 genotype in the lung (*p* = 1.00 × 10^-9^).

**Discussion**

In this study, we analyzed the association of *COL6A4P2* gene polymorphisms with susceptibility to LC. We identified that rs34445363 in *COL6A4P2* was associated with an increased risk of LC. Our results also suggested that rs34445363 site mutations increase the risk of LUAD, while the mutation of rs61733464 significantly decreased the LUAD risk. These results suggest an association between genetic polymorphisms of *COL6A4P2* and susceptibility to LC.

Numerous studies have shown that collagen levels play an essential role in the development of LC ^20, 21^. Naveen *et al.* ^22^ identified collagen VI as a potential biomarker for the early diagnosis of LC by proteomic analysis, suggesting that LC is associated with collagen-encoding genes. The *COL6A4P2* gene is a pseudogene formed by the chromosomal break of the collagen-encoding gene *COL6A4* ^13, 23^; therefore, we speculate that the *COL6A4P2* gene may be associated with LC. Our results suggest that the rs34445363 mutation in the *COL6A4P2* gene significantly increases the risk of LC, validating our conjecture, and is consistent with previous studies.

Our results also found that the association between *COL6A4P2* gene polymorphism and LC risk was influenced by gender and age. A retrospective analysis by Oh *et al.* ^24^ assessed the crucial effects of sex and age on the development of LC. Aareleid *et al.* ^25^ revealed that LC has different incidence rates in different genders and ages. These studies are consistent with our results and enhance the credibility of our findings.

Furthermore, we predicted the differential expression of *COL6A4P2* in normal lung tissues and LC tissues using a database. Voiles *et al.* ^12^ found that collagen VI protein levels increased in tumor lung tissue and speculated that the expression of the *COL6A4P2* gene in tumor lung tissue is variable. This is consistent with our predictions. Fagerberg *et al.* ^26^ found that the *COL6A4P2* gene is specifically expressed in human lung tissue by genome-wide integration analysis of transcriptomics and antibody proteomics. These findings suggest the important research significance of *the COL6A4P2* gene in the development of LC, prompting that the *COL6A4P2* gene deserves further study.

**Conclusion**

In conclusion, the present study is the first to investigate the association between *COL6A4P2* and LC. Our findings indicated that *COL6A4P2* gene polymorphism is associated with LC risk in the Chinese Han population. However, it is necessary to conduct further studies in other races and larger sample sizes to confirm our results. Our study provides a basic reference for individualized LC prevention.

**Acknowledgments**

We thank all authors for their contributions and supports. We are also grateful to all participants for providing blood samples.

**References**

1. Kou Y, Li G, Shao J, et al. Genome-Wide Profiling Reveals That Herbal Medicine Jinfukang-Induced Polyadenylation Alteration Is Involved in Anti-Lung Cancer Activity. Evid Based Complement Alternat Med. 2017;2017:5326909. doi:10.1155/2017/5326909

2. Albarqouni L, Elessi K, Abu-Rmeileh NME. A comparison between health research output and burden of disease in Arab countries: evidence from Palestine. Health Res Policy Syst. Mar 15 2018;16(1):25. doi:10.1186/s12961-018-0302-4

3. de Groot PM, Wu CC, Carter BW, Munden RF. The epidemiology of lung cancer. Transl Lung Cancer Res. Jun 2018;7(3):220-233. doi:10.21037/tlcr.2018.05.06

4. Sun KX, Zheng RS, Zeng HM, et al. [The incidence and mortality of lung cancer in China, 2014]. Zhonghua Zhong Liu Za Zhi. Nov 23 2018;40(11):805-811. doi:10.3760/cma.j.issn.0253-3766.2018.11.002

5. Bhimji SS, Wallen JM. Cancer, Lung, Adenocarcinoma. StatPearls. 2018.

6. Cao M, Chen W. Epidemiology of lung cancer in China. Thorac Cancer. Nov 28 2018;doi:10.1111/1759-7714.12916

7. Mohamed S, Bayoumi H, El-Aziz NA, Mousa E, Gamal Y. Prevalence, risk factors, and impact of lung Cancer on outcomes of idiopathic pulmonary fibrosis: a study from the Middle East. Multidiscip Respir Med. 2018;13:37. doi:10.1186/s40248-018-0150-7

8. Liu D, Wen H, He J, et al. Society for Translational Medicine Expert Consensus on the preoperative assessment of circulatory and cardiac functions and criteria for the assessment of risk factors in patients with lung cancer. J Thorac Dis. Sep 2018;10(9):5545-5549. doi:10.21037/jtd.2018.08.91

9. Li H, Yang Z, Li J, et al. Genetic variants in lncRNA HOTAIR are associated with lung cancer susceptibility in a Chinese Han population in China: a case-control study. Cancer Manag Res. 2018;10:5209-5218. doi:10.2147/CMAR.S175961

10. Dimitrakopoulos FD, Antonacopoulou AG, Kottorou AE, et al. NF-kB2 Genetic Variations are Significantly Associated with Non-Small Cell Lung Cancer Risk and Overall Survival. Sci Rep. Mar 27 2018;8(1):5259. doi:10.1038/s41598-018-23324-3

11. Chiu KH, Chang YH, Wu YS, Lee SH, Liao PC. Quantitative secretome analysis reveals that COL6A1 is a metastasis-associated protein using stacking gel-aided purification combined with iTRAQ labeling. J Proteome Res. Mar 4 2011;10(3):1110-25. doi:10.1021/pr1008724

12. Voiles L, Lewis DE, Han L, et al. Overexpression of type VI collagen in neoplastic lung tissues. Oncol Rep. Nov 2014;32(5):1897-904. doi:10.3892/or.2014.3438

13. Fitzgerald J, Rich C, Zhou FH, Hansen U. Three novel collagen VI chains, alpha4(VI), alpha5(VI), and alpha6(VI). J Biol Chem. Jul 18 2008;283(29):20170-80. doi:10.1074/jbc.M710139200

14. Wu C, Wei Y, Zhu Y, et al. Identification of cancer-related potential biomarkers based on lncRNA-pseudogene-mRNA competitive networks. FEBS Lett. Mar 2018;592(6):973-986. doi:10.1002/1873-3468.13011

15. Lynn H, Sun X, Ayshiev D, et al. Single nucleotide polymorphisms in the MYLKP1 pseudogene are associated with increased colon cancer risk in African Americans. PLoS One. 2018;13(8):e0200916. doi:10.1371/journal.pone.0200916

16. Wei CC, Nie FQ, Jiang LL, et al. The pseudogene DUXAP10 promotes an aggressive phenotype through binding with LSD1 and repressing LATS2 and RRAD in non small cell lung cancer. Oncotarget. Jan 17 2017;8(3):5233-5246. doi:10.18632/oncotarget.14125

17. Huang CY, Xun XJ, Wang AJ, et al. CHRNA5 polymorphisms and risk of lung cancer in Chinese Han smokers. Am J Cancer Res. 2015;5(10):3241-8.

18. Dai ZJ, Liu XH, Ma YF, et al. Association Between Single Nucleotide Polymorphisms in DNA Polymerase Kappa Gene and Breast Cancer Risk in Chinese Han Population: A STROBE-Compliant Observational Study. Medicine (Baltimore). Jan 2016;95(2):e2466. doi:10.1097/md.0000000000002466

19. He J, Zou Y, Liu X, et al. Association of Common Genetic Variants in Pre-microRNAs and Neuroblastoma Susceptibility: A Two-Center Study in Chinese Children. Molecular therapy Nucleic acids. Jun 1 2018;11:1-8. doi:10.1016/j.omtn.2018.01.003

20. Shea MP, O'Leary KA, Wegner KA, Vezina CM, Schuler LA. High collagen density augments mTOR-dependent cancer stem cells in ERalpha+ mammary carcinomas, and increases mTOR-independent lung metastases. Cancer Lett. Oct 1 2018;433:1-9. doi:10.1016/j.canlet.2018.06.025

21. Li Y, Chen Y, Ma Y, Nenkov M, Haase D, Petersen I. Collagen prolyl hydroxylase 3 has a tumor suppressive activity in human lung cancer. Exp Cell Res. Feb 1 2018;363(1):121-128. doi:10.1016/j.yexcr.2017.12.020

22. Chaudhary N, Bhatnagar S, Malik S, Katare DP, Jain SK. Proteomic analysis of differentially expressed proteins in lung cancer in Wistar rats using NNK as an inducer. Chem Biol Interact. Jul 5 2013;204(2):125-34. doi:10.1016/j.cbi.2013.05.004

23. Gara SK, Grumati P, Urciuolo A, et al. Three novel collagen VI chains with high homology to the alpha3 chain. J Biol Chem. Apr 18 2008;283(16):10658-70. doi:10.1074/jbc.M709540200

24. Oh JY, Lee YS, Min KH, et al. Presence of lung cancer and high gender, age, and physiology score as predictors of acute exacerbation in combined pulmonary fibrosis and emphysema: A retrospective study. Medicine (Baltimore). Aug 2018;97(31):e11683. doi:10.1097/MD.0000000000011683

25. Aareleid T, Zimmermann ML, Baburin A, Innos K. Divergent trends in lung cancer incidence by gender, age and histological type in Estonia: a nationwide population-based study. BMC Cancer. Aug 30 2017;17(1):596. doi:10.1186/s12885-017-3605-x

26. Fagerberg L, Hallstrom BM, Oksvold P, et al. Analysis of the human tissue-specific expression by genome-wide integration of transcriptomics and antibody-based proteomics. Mol Cell Proteomics. Feb 2014;13(2):397-406. doi:10.1074/mcp.M113.035600

**Supporting informationS1 Table. Association between *COL6A4P2* polymorphism and lymph node metastasis status in patients with lung cancer.**

**S2 Table. FPRP and statistical power values of the association analysis results in the subgroup analysis.**

**S3 Table. Haplotype frequencies of *COL6A4P2* polymorphisms and their association with the risk of lung cancer.**

**S4 Table. Genotyping results of all participants.**

|  | |  |  |
| --- | --- | --- | --- |
|  |  |  |  |
|  |  |  |  |
|  |  |  |  |
|  |  |  |  |
|  |  |  |  |
|  |  |  |  |
|  |  |  |  |
|  |  |  |  |
|  |  |  |  |
|  |  |  |  |
|  |  |  |  |
|  |  |  |  |
|  |  |  |  |
|  |  |  |  |

|  |  |  |  |  | |  |  |  |  |
| --- | --- | --- | --- | --- | --- | --- | --- | --- | --- |
|  |  |  |  |  |  |  |  |  |  |
|  |  |  |  |  |  |  |  |  |  |
|  |  |  |  |  |  |  |  |  |  |
|  |  |  |  |  |  |  |  |  |  |
|  |  |  |  |  |  |  |  |  |  |
|  |  |  |  |  |  |  |  |  |  |

|  |  |  |  |  | | |  | | |  | | |
| --- | --- | --- | --- | --- | --- | --- | --- | --- | --- | --- | --- | --- |
|  |  |  |  |  |  |  |  |  |  |  |  |  |
|  |  |  |  |  |  |  |  |  |  |  |  |  |
|  |  |  |  |  |  |  |  |  |  |  |  |  |
|  |  |  |  |  |  |  |  |  |  |  |  |  |
|  |  |  |  |  |  |  |  |  |  |  |  |  |
|  |  |  |  |  |  |  |  |  |  |  |  |  |
|  |  |  |  |  |  |  |  |  |  |  |  |  |
|  |  |  |  |  |  |  |  |  |  |  |  |  |
|  |  |  |  |  |  |  |  |  |  |  |  |  |
|  |  |  |  |  |  |  |  |  |  |  |  |  |
|  |  |  |  |  |  |  |  |  |  |  |  |  |
|  |  |  |  |  |  |  |  |  |  |  |  |  |
|  |  |  |  |  |  |  |  |  |  |  |  |  |
|  |  |  |  |  |  |  |  |  |  |  |  |  |
|  |  |  |  |  |  |  |  |  |  |  |  |  |
|  |  |  |  |  |  |  |  |  |  |  |  |  |
|  |  |  |  |  |  |  |  |  |  |  |  |  |

|  |  |  |  |  |  |  |
| --- | --- | --- | --- | --- | --- | --- |
|  |  |  |  |  |  |  |
|  |  |  |  |  |  |  |
|  |  |  |  |  |  |  |
|  |  |  |  |  |  |  |
|  |  |  |  |  |  |  |
|  |  |  |  |  |  |  |
|  |  |  |  |  |  |  |
|  |  |  |  |  |  |  |

|  |  | |  | |  | | | | | | |  | | | | | | | |  |
| --- | --- | --- | --- | --- | --- | --- | --- | --- | --- | --- | --- | --- | --- | --- | --- | --- | --- | --- | --- | --- |
|  |  |  |  |  |  | |  |  | |  | |  | |  | |  | |  | |  |
|  |  | |  | |  | |  |  | |  | |  | |  | |  | |  | |  |
|  |  | |  | |  | |  |  | |  | |  | |  | |  | |  | |  |
|  |  | |  | |  | |  |  | |  | |  | |  | |  | |  | |  |
|  |  | |  | |  | |  |  | |  | |  | |  | |  | |  | |  |
|  |  | |  | |  | |  |  | |  | |  | |  | |  | |  | |  |
|  |  | |  | |  | |  |  | |  | |  | |  | |  | |  | |  |
|  |  | |  | |  | |  |  | |  | |  | |  | |  | |  | |  |
|  |  | |  | |  | |  |  | |  | |  | |  | |  | |  | |  |
|  |  | |  | |  | |  |  | |  | |  | |  | |  | |  | |  |
|  |  | |  | |  | |  |  | |  | |  | |  | |  | |  | |  |
|  |  | |  | |  | |  |  | |  | |  | |  | |  | |  | |  |
|  |  | |  | |  | |  |  | |  | |  | |  | |  | |  | |  |
|  |  | |  | |  | |  |  | |  | |  | |  | |  | |  | |  |
|  |  | |  | |  | |  |  | |  | |  | |  | |  | |  | |  |
|  |  | |  | |  | |  |  | |  | |  | |  | |  | |  | |  |
|  |  | |  | |  | |  |  | |  | |  | |  | |  | |  | |  |
|  |  |  | |  | | | | | | | | |  | | | | | | | |
|  |  |  |  |  | |  | | |  | |  | |  | |  | |  | |  | |
|  |  |  | |  | |  | | |  | |  | |  | |  | |  | |  | |
|  |  |  | |  | |  | | |  | |  | |  | |  | |  | |  | |
|  |  |  | |  | |  | | |  | |  | |  | |  | |  | |  | |
|  |  |  | |  | |  | | |  | |  | |  | |  | |  | |  | |
|  |  |  | |  | |  | | |  | |  | |  | |  | |  | |  | |
|  |  |  | |  | |  | | |  | |  | |  | |  | |  | |  | |
|  |  |  | |  | |  | | |  | |  | |  | |  | |  | |  | |
|  |  |  | |  | |  | | |  | |  | |  | |  | |  | |  | |
|  |  |  | |  | |  | | |  | |  | |  | |  | |  | |  | |
|  |  |  | |  | |  | | |  | |  | |  | |  | |  | |  | |
|  |  |  | |  | |  | | |  | |  | |  | |  | |  | |  | |
|  |  |  | |  | |  | | |  | |  | |  | |  | |  | |  | |
|  |  |  | |  | |  | | |  | |  | |  | |  | |  | |  | |
|  |  |  | |  | |  | | |  | |  | |  | |  | |  | |  | |
|  |  |  | |  | |  | | |  | |  | |  | |  | |  | |  | |
|  |  |  | |  | |  | | |  | |  | |  | |  | |  | |  | |
